# The effectiveness and safety of acupuncture for allergic rhinitis: Protocol for an overview of systematic reviews and meta-analyses

**DOI:** 10.1097/MD.0000000000021225

**Published:** 2020-07-17

**Authors:** Jun Yang, Jun Xiong, Xue Wang, Ting Yuan, Yong Fu, Yunfeng Jiang, Xiaohong Zhou, Kai Liao, Lingling Xu

**Affiliations:** a Jiangxi University of TCM; b The Affiliated Hospital of Jiangxi University of TCM, Nanchang, Jiangxi, China.

**Keywords:** acupuncture, allergic rhinitis, overview, protocol

## Abstract

**Background:**

Allergic rhinitis (AR) is one of the most common allergic disorders globally. Several systematic reviews and meta-analyses have reported the effectiveness of acupuncture for patients with AR. However, the evidence has not been systematically synthesized. This overview aims to map, synthesize, and assess the reliability of evidence generated from these systematic reviews (SRs) and meta-analyses of acupuncture for AR.

**Methods:**

A Comprehensive literature search will be conducted through the PubMed, Embase, the Cochrane Library of Systematic Reviews, the China National Knowledge Infrastructure Database, Wanfang Database, Chinese Biomedical Literature Database, and Chinese Scientific Journal Database from inception until January 2020. Additionally, the PROSPERO database and the reference list of included studies will be searched for unpublished, ongoing, or recently completed SRs and meta-analyses. The reviewers will identify reviews independently and extract data according to the methodological guidelines for overviews provided by the Cochrane Collaboration. The risk of bias will be assessed based on the Risk of Bias in Systematic Reviews. The methodological and reporting quality of the included reviews will be assessed using the Assessing the Methodological Quality of Systematic Reviews (V.2) tool and the Preferred Reporting Items for Systematic Review and Meta-Analyses statement. The outcomes of interest include total nasal symptom score, rhinoconjunctivitis quality-of-life questionnaire, immunoglobulin E, visual analog scale, laboratory examination, and side effects. The quality of evidence of outcomes will be evaluated using the Grading of Recommendations Assessment, Development and Evaluation. The evidence will be synthesized where appropriate based on patient subgroups and outcomes.

**Ethics and dissemination:**

Ethical approval is not required for overviews. We plan to publish results in peer-reviewed journals and present at international and national academic, clinical, and patient conferences.

**Results:**

The results will be published in a peer-reviewed journal.

**Conclusion:**

This overview will provide comprehensive evidence of acupuncture for patients with AR.

**PROSPERO registration number:**

CRD42019140756.

## 1 Introduction

### 1.1 Description of the condition

Allergic rhinitis (AR) is a prevalent noninfectious inflammatory disorder of the nasal mucosa, which triggers mainly immunoglobulin E (IgE) response after people are exposed to different allergens. Clinically, nasal obstruction, frequent sneezing, runny nose, itchy and watery eyes are the main manifestations, accompanied by some loss of sense.^[1,2]^ In some cases, these conditions severely affect the quality of life.^[3–5]^

People of all ages may develop AR, while approximately 80% of individuals diagnosed with AR develop symptoms before the age of 20 years. Further, 13% of children in the US and 15.79% in China were diagnosed with AR.^[6,7]^ Ten years ago, 40% of the worlds population was affected, and the prevalence of AR in Europe was 23% to 30%.^[8–10]^ A survey conducted in 2005 reported that almost 800 million people suffered from the disease in the rural areas of China.^[7]^ The prevalence of AR continues to increase annually across many countries.^[3]^

An economic survey showed that they spent more money on medication costs and health care in a family of patients with AR.^[11]^ Extra expenses increase the financial burden on the family. In the meantime, AR increases the risk of other diseases such as headache, upper airway cough syndrome, hypomnesis, chronic rhinosinusitis, and asthma. AR is an independent risk factor for the development of asthma. According to a survey conducted in the US, 78% of patients with asthma have AR.^[12]^

The therapeutic principle of AR involves a comprehensive approach including environmental control, pharmacotherapy, immunotherapy, and patient education.^[8]^ Antihistamines^[13,14]^ and intranasal corticosteroid spray^[15]^ serve as the first-line therapy for AR through blocking multiple inflammatory pathways to alleviate the painful sufferings and nasal symptoms. However, the disease recurrence makes medication less effective over time. Intranasal anticholinergics are recommended as the second-line therapy for AR by inhibiting both watery secretion of nasal glands and vasodilatation of airway blood vessels.^[16]^

### 1.2 Description of the intervention

Acupuncture, which is as old as 2600 BC, refers to a practice in which fine needles were inserted into patients specific exterior body locations or acupoints to relieve their suffering. Acupuncture includes electropuncture, ignipuncture, acupoint injection, and the use of an auricular needle.

Acupuncture has been widely accepted for various otolaryngologic disorders. It not only helps improve the blood indices with an increased volume of blood flow but also regulates the immunologic function of the human body, thus exerting therapeutic effects against AR.^[17]^ Some studies reported that acupoint injection at “Yingxiang” (LI 20) and “Yintang” (GV 29) might relieve nasal allergic symptoms possibly by downregulating the expression of histamine receptors H1 and H4 in the nasal mucosa of rats with AR or upregulating the level of nasal mucosal Foxp3 and suppressing the levels of nasal RORyt and serum IL-17 to correct the imbalance of Th17/Treg.^[18,19]^ Recently, some acupuncturists penetrated the needle at 1 special acupoint to reach the sphenopalatine ganglion and achieve a definite effect.^[20]^ The latest American Clinical Practice Guideline for AR, published in 2015, recommends that acupuncture be offered as an option for patients with interest in nonpharmacologic approaches to manage AR.^[21,22]^

### 1.3 The reason to perform this overview

Recently, the number of SRs and meta-analyses has increased. An increasing number of studies reported the effectiveness and safety of acupuncture for AR. Well-conducted SRs or meta-analyses have been recognized as the basis for evaluating clinical efficacy and formulating clinical guidelines.^[22,23]^ High-quality studies can provide the best evidence for clinical practice and health-care decisions. Overviews of SRs and meta-analyses compile information from multiple SRs and meta-analyses for a comprehensive synthesis of evidence. The more the information collected, the better the quality of evidence provided for clinical work.^[24]^

Considering previous findings, Zhou Jun conducted an overview of the use of acupuncture in treating AR.^[25]^ He evaluated the evidence using Assessing the Methodological Quality of Systematic Reviews (AMSTAR) and Grading of Recommendations Assessment, Development and Evaluation (GRADE). The AMSTAR-2 has been updated. However, the reporting quality of the evidence could not be evaluated without making a quantitative analysis of the outcomes. To overcome previous limitations, the overview will be conducted to provide a comprehensive summary of the evidence.

### 1.4 Description of the objectives

The objectives of this study are as follows:

1. Explore the methodological and reporting quality of available SRs using AMSTAR-2 and Preferred Reporting Items for Systematic Reviews and Meta-analyses (PRISMA)
2. Summarize the best current evidence for the effectiveness and safety of acupuncture for AR through qualitative analysis of the outcomes.
3. Provide more reliable, evidence-based medical references for clinical practitioners and researchers.

## 2 Methods and analysis

### 2.1 Protocol and registration

This overview protocol has been registered on PROSPERO as CRD42019140756, and is available from: https://www.crd.york.ac.uk/prospero/display_record.php?RecordID=140756. This overview will be reported according to the Preferred Reporting Items for Systematic Reviews and Meta-analyses Protocols (PRISMA-P).^[26]^ The changes in the full review will be described if needed.

### 2.2 Eligibility criteria

#### 2.2.1 Type of studies

All relevant SRs and meta-analyses of randomized clinical trials published in English and Chinese about acupuncture for AR will be included.

#### 2.2.2 Type of participants

Study participants of different age ranges with all types of AR will be included, regardless of sex, race, occupation, education, nationality, etiology, and severity.

#### 2.2.3 Type of interventions

Acupuncture (it includes body acupuncture, electroacupuncture, fire acupuncture, acupoint injection, and ear acupuncture) will be included as a single intervention or major part of a combination therapy with other active interventions (e.g., western medicine, moxibustion, or cupping).

#### 2.2.4 Type of comparators

The comparative interventions will be sham acupuncture, placebo, no treatment, or other active treatments. The following treatment comparisons will be performed:

1. Acupuncture vs no treatment
2. Acupuncture vs placebo or sham acupuncture
3. Acupuncture vs other active therapies
4. Acupuncture + active therapy vs the same active therapy.

#### 2.2.5 Types of outcome measurements

*Primary outcomes:* The total nasal symptom score (TNSS)^[27]^ will be recommended as the primary outcome, and the score will be evaluated according to the degree of 4 nasal symptoms: sneezing, rhinorrhea, nasal itching, and nasal obstruction. Also, the score change before and after treatment will be compared at least for 1 treatment cycle.

*Secondary outcomes:* The secondary outcomes in this review will include mainly the following aspects:

1. Rhinoconjunctivitis quality–life questionnaire (RQLQ)^[28]^
2. Allergen detection: IgE
3. Side effects or adverse events
4. Visual analog scale (VAS): Patients grade their symptoms by putting a vertical line on a 1- to 10-cm line representing the severity score from 0 “no symptoms” to 10 “highest level of symptoms”
5. Laboratory examination: immunoglobulin A (IgA), IgM, IgG, C3, and C4

### 2.3 Information sources and search strategy

SRs and meta-analyses will be identified through systematic searches of the following databases: PubMed, Embase, the Cochrane Library of Systematic Reviews, the China National Knowledge Infrastructure Database, Wanfang Database, Chinese Biomedical Literature (CBM) Database, and Chinese Scientific Journal Database (VIP) from inception until January 2020. The language will be restricted to Chinese and English. To ensure comprehensiveness of the study, the PROSPERO database and the reference lists of included studies will be searched for unpublished, ongoing, or recently completed SRs. A comprehensive search strategy will be conducted, comprising free text and vocabulary terms identified by the expert consultation group from our preliminary searches of SRs on acupuncture for AR. The preliminary search strategy for PubMed is presented in Table 1.

**Table 1:**
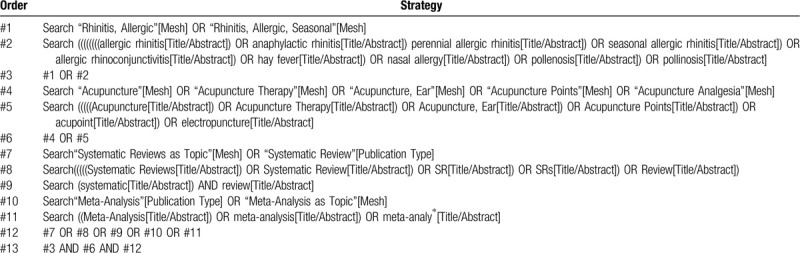
Search strategy (PubMed).

### 2.4 Studies selection

Studies will be identified using NoteExpress 3.0. After the initial removal of duplicate studies, 2 reviewers (TY and XW) will independently screen titles and abstracts based on the eligibility criteria. Full-text studies will be retrieved for all potentially includable SRs or SR protocols. If studies contain insufficient information to make a decision about eligibility, JY will try to contact authors of the original reports to obtain further details. The exclusion criteria of this overview will include mainly the following aspects:

1. Patients with asthma, nasosinusitis, vasomotor rhinitis, infectious rhinitis, or hormonal rhinitis
2. Repeated publications and studies lacking the required test data
3. Full text not obtained through various approaches

**Figure 1:**
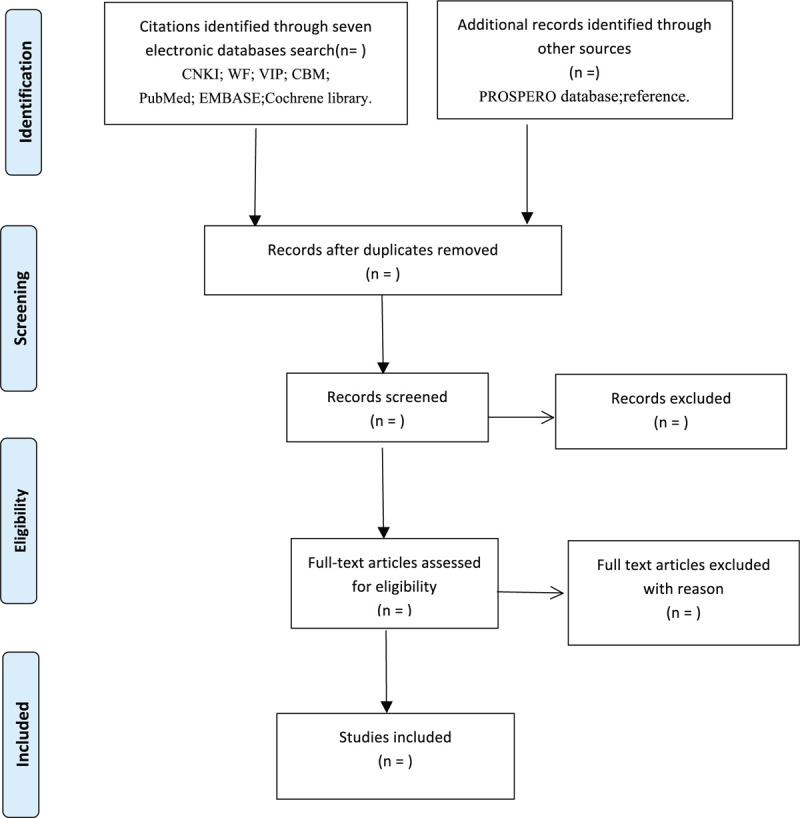
Flowchart of literature selection.

During the procedure, disagreements will be resolved by discussion or consensus with the third reviewer (FYJ). Study selection will be performed in accordance with the PRISMA^[29]^ flowchart (Fig. 1).

### 2.5 Data extraction

Two reviewers (XHZ and LLX) will independently extract the following information from each included study:

1. Bibliographic details (author and publication year)
2. Methodological characteristics (information sources, search end date, eligibility criteria, publication date range of included studies, intervention(s), comparison(s), acupoint, dose of the dug, length of follow-up, method of pooling and bias assessment, and funding)
3. Patient characteristics (age, sex, and type of AR)
4. Results [number of studies included in the meta-estimate, event rate in exposed and unexposed trial arms or patient populations, meta-estimate, risk of bias within included studies, adverse effects, and 95% confidence interval (CI)].

If the reported data are not sufficient, JY will try to contact the authors for further information to supplement the missing data. Any disagreements will be resolved by discussion or consensus with the third reviewer (KL). The data extracted will be recorded in WPS 2019.

### 2.6 Data analysis

#### 2.6.1 Assessment of risk of bias

Two reviewers (TY and XW) will independently assess the risk of bias of included studies using the Risk of Bias in Systematic reviews (ROBIS) tool.^[30]^ It has 3 phases: assess relevance (optional), identify concerns with the review process, and judge the risk of bias. Phase 2 covers 4 domains (“study eligibility criteria,” “identification and selection of studies,” “data collection and study appraisal,” and “synthesis and findings”). Phase 3 assesses the overall risk of bias of review findings.^[30]^ The results of each domain and phase 3 will be categorized as low (meet all criteria), unclear (trials with insufficient information to judge), and high risk (meet none of the criteria) of bias. The consensus will be reached through discussion between 2 reviewers or an independent decision obtained from the expert (JX), if necessary.

#### 2.6.2 Assessment of methodological and reporting quality

Two reviewers (JY and XHZ) will, respectively, assess the methodological and reporting quality of included studies on the basis of the AMSTAR-2^[31]^ and PRISMA. Disagreement will be solved through discussion between 2 reviewers or an independent decision obtained from the expert (YFJ), if necessary.

The number and 95% confidence intervals of 3 levels (“Yes,” “No,” or “Partial Yes”) related to the AMSTAR-2 and PRISMA items will be calculated. In the meantime, the 3 levels will be scored as 1, 0.5, or 0 points for statistical analysis. The AMSTAR-2 and PRISMA score percentage will be rated as follows: very poor (<30%), poor (30%–50%), fair (50%–70%), good (70%–90%), and excellent (>90%).

1. AMSTAR-2: It has 16 items, of which 7 are critical domains. Each checklist item will be assessed as “Yes,” “No,” or “Partial Yes” to indicate the degree of compliance.
2. PRISMA: It is a 27-items checklist. Each checklist item will be assessed as “Yes,” “No,” or “Partial Yes” to indicate the degree of compliance.

#### 2.6.3 Quality of the evidence (GRADE)

The quality of evidence will be evaluated using the GRADE approach.^[32]^ The evidence quality of all outcomes will be assessed as 4 levels (high, moderate, low, or very low) from 5 aspects (limitations, inconsistency, indirectness, imprecision, and publication bias). Two reviewers (KL and LLX) will conduct the assessment process separately and describe the reason for downgraded or upgraded outcomes affecting the quality of evidence to guarantee the reliability and transparency of results. Any disagreement will be resolved through discussion and consultation with a third author (YFJ) until a consensus is reached.

#### 2.6.4 Dealing with missing data

If the published SRs and meta-analyses lack specific data, attempts will be made to contact the original authors and obtain the missing information. When the data provided are insufficient, the analysis will rely on available data, and the potential impact of missing data will be discussed.

### 2.7 Data synthesis

Before data synthesis, some overlapping primary studies in the included SRs and meta-analyses should be considered. Different measures will be taken for different situations of the overlapping primary studies: if the primary studies are completely overlapping, the highest-quality review will be selected. If the primary studies partially overlap, both reviews will be retained when the lower-quality review consists of more than one-third new studies. If the primary studies do not overlap, both reviews will be retained. The quality of the review will be comprehensively assessed using ROBIS and AMSTAR-2.

If the result is reported differently in reviews, the relative risks or odds ratios will be standardized for dichotomous outcomes and mean difference or standard mean difference for continuous outcomes using the equations published in the Cochrane Handbook for Systematic Reviews of Interventions.^[33]^ RevMan5.3.5 software will be used to calculate the standardized effect. The random-effects model (*I*^2^ ≥ 50%) or fixed-effects model (*I*^2^ < 50%) will be selected according to the heterogeneity levels of the included SRs and meta-analyses. If the *I*^2^ value is higher than 75%, the clinical or methodological heterogeneity will be explored through discussion with the review team. When the meta-analysis is not possible, a narrative analysis will be performed. Indirect comparisons of different acupuncture therapies will also be conducted using relative effectiveness outcomes including relative sensitivity and relative specificity.

### 2.8 Subgroup analysis and investigation of heterogeneity

If considerable heterogeneity is observed in the included SRs and meta-analyses, a subgroup analysis or meta-regression of separate review data will be performed, grouped by differences in the scope of the review, including the type of AR (perennial AR and seasonal AR), age (children and adult), length of the following intervention (1 month, 2 months, and 3 months), and the frequency of the intervention.

### 2.9 Publication bias

The publication bias will be assessed using a funnel plot when the included systematic reviews per outcome (TNSS, RQLQ, IgE, VAS, laboratory examination, and side effects) is more than 10.

### 2.10 Ethics and dissemination

Ethical approvals and patient consent are not necessary because this overview will be based on published systematic reviews and meta-analyses for no personal data involved. The results of the overview will be disseminated through peer-reviewed publications according to the PRISMA-P guidelines.

## 3 Discussion

In the discussion section, the following subsections will be included:

1. summary of main findings;
2. strength and limitations;
3. comparison with other studies and opinions;
4. interpretation of results;
5. ethical considerations and funding; and
6. conclusion.

AR is one of the most common allergic disorders globally, affecting 10% to 40% of the world population.^[34]^ Many people may resort to medicine to relieve the undesirable symptoms of AR. However, the most common adverse events associated with the use of intranasal formulations include nasal burning, poor taste, sedation, more frequent dosing, and increased cost compared with oral formulations. Doctors should pay more attention to side effects with the initiation of intranasal antihistamines.

In traditional Chinese medicine, AR belongs to the category of “Bi Qiu.” The cause of AR may be deficiency, excess, cold, or heat, involving the “Zang-Fu” organs of the lungs, spleen, and kidneys on the basis of the concept of traditional Chinese medicine. Many methods are available to help patients improve their undesirable symptoms. Acupuncture is a specific therapy based on the concept of traditional Chinese medicine. It can increase blood volume, increase the blood flow, and regulate the immunologic function of patients with AR.

Despite some SRs and meta-analyses about acupuncture for AR, no relative overview was conducted to investigate the effectiveness and safety of acupuncture for AR. Hence, the methodological and reporting quality of these studies remains unclear. This overview will be conducted systematically to assess the current evidence.

To comprehensively assess the SRs and meta-analyses, the evidence about acupuncture for AR will be collected from domestic and abroad databases by searching the unpublished, ongoing, or recently completed SRs in the PROSPERO database and the reference list of included studies. Moreover, a scientific search strategy will be designed by consulting some experts and referencing to the preliminary searches of SRs on acupuncture for AR. The included studies will be assessed using several appraisal instruments, such as AMSTAR 2, ROBIS, and PRISMA, to obtain some reliable and credible recommendations for clinical and further studies.

This study may have some limitations. First, some overlapping primary studies among the included SRs and meta-analyses cannot be ignored. If the overlapping studies are not processed properly, the duplicate clinical trials may affect the synthetic result. Second, some information may be missing due to the inclusion of studies written only in English and Chinese.

The study is expected to provide a better approach for the treatment of AR and provide reliable evidence. Furthermore, the overview may encourage further research to improve the limitations.

## Author contributions

**Conceptualization:** Jun Yang, Yong Fu.

**Data curation:** Xue Wang, Ting Yuan.

**Formal analysis:** Hong xiao Zhou, Ling ling Xu.

**Investigation:** Jun Yang, Feng yun Jiang.

**Methodology:** Xue Wang, Ting Yuan.

**Software:** Hong xiao Zhou, Ling ling Xu.

**Supervision:** Feng yun Jiang, Kai Liao.

**Writing – original draft:** Jun Yang, Xue Wang, Ting Yuan.

**Writing – review & editing:** Yong Fu, Feng yun Jiang, Hong xiao Zhou, Kai Liao, Ling ling Xu.
